# Hypokalemia Requiring Hospitalization After Taking Semaglutide Injections

**DOI:** 10.7759/cureus.87382

**Published:** 2025-07-06

**Authors:** Krista N Grennan, Devina Singh, Ana-Maria Chindris, Jessica R Wilson

**Affiliations:** 1 Internal Medicine, Mayo Clinic, Jacksonville, USA; 2 Hospital Medicine, Hospital Corporation of America Florida Lake City Medical Center, Lake City, USA; 3 Endocrinology, Diabetes, and Metabolism, Mayo Clinic, Jacksonville, USA

**Keywords:** diabetes type 2, drug-induced hypokalemia, glp-1 receptor agonists, hypokalemia, obesity, weight loss and obesity, weight-loss intervention

## Abstract

Glucagon-like peptide-1 receptor agonists are not known to cause hypokalemia. We report two patients taking semaglutide who developed hypokalemia requiring hospitalization. Patient one was a 48-year-old female with type 2 diabetes who presented to the clinic for diabetes management. She was prescribed injectable semaglutide for glucose control. Six months later, she developed emesis and nausea with potassium levels of 2.7 mmol/L, requiring hospitalization for potassium replacement. A second readmission occurred several months later for hypokalemia of 2.6 mmol/L. She required peripheral parenteral nutrition and was transitioned to a feeding tube. Semaglutide was discontinued, and insulin was initiated with resolution of hypokalemia. Patient two was a 45-year-old female with obesity and prediabetes who presented to the clinic for treatment of obesity and was prescribed semaglutide for weight loss. Pre-semaglutide serum potassium levels were 3.4 mmol/L while taking a stable dose of 50 mg of chlorthalidone daily and 20 mEq of potassium daily. After 1.5 months on semaglutide, the patient's potassium decreased to 3.1 mmol/L. Repeat potassium five days later was 2.5 mmol/L, which prompted hospital admission. Chlorthalidone was discontinued, and potassium was increased to 40 mEq daily. Her potassium improved to 4.2 mmol/L. Semaglutide was eventually discontinued. She stopped potassium supplementation. Her potassium has remained stable at or above 3.8 mmol/L. We report the hospitalization of two patients for hypokalemia during their semaglutide treatment. Hypokalemia is not a reported side effect, and the cause in these patients may be multifactorial. This potential side effect should be monitored in patients who are at risk of hypokalemia.

## Introduction

Semaglutide, a glucagon-like peptide-1 receptor agonist (GLP-1RA), has been approved by the United States Food and Drug Administration for the treatment of patients with type 2 diabetes mellitus and for weight loss in patients with obesity [1]. GLP-1RAs, via the incretin pathway, stimulate the release of insulin as well as decrease the release of glucagon [2]. They improve glycemic control with low risk for hypoglycemia [2].

Multiple studies have demonstrated that GLP-1RAs aid in controlling diabetes and promoting weight loss. The Semaglutide Unabated Sustainability in Treatment of Type 2 Diabetes (SUSTAIN) phase 3 clinical trials, involving over 8,000 patients, demonstrated that the semaglutide treatment group was associated with increased weight loss and sustained improvement in glycemic control [2]. The American Diabetes Association (ADA) also identifies semaglutide as one of the medications in the "very high efficacy" category for weight loss and for having a greater likelihood of achieving glycemic control, which is the highest efficacy category in their Standards of Care in Diabetes 2024 guidelines [3]. Similarly, the American Association of Clinical Endocrinology's (AACE) 2023 Consensus Statement on type 2 diabetes management emphasizes that GLP-1RAs are preferred medications in patients who are classified as overweight or obese, have a risk of hypoglycemia, and/or have severe hyperglycemia [4].

In addition to semaglutide's well-demonstrated benefits for weight loss and diabetes, it has also been found to be beneficial in patients with cardiovascular disease [3]. The SUSTAIN phase 3 clinical trials also demonstrated a significant reduction in the incidence of cardiovascular events compared to the current standard of care [2]. The ADA also recommends the use of a GLP-1RA and/or a sodium-glucose cotransporter-2 inhibitor independent of metformin use in patients with known chronic kidney disease, cardiovascular disease, or risk factors for cardiovascular disease [3]. Given these demonstrated benefits, semaglutide has become a popular diabetes and obesity drug.

Semaglutide is mainly associated with gastrointestinal adverse effects, including nausea, emesis, and diarrhea [2,5]. There are also rarer and potentially fatal side effects, including pancreatitis, severe gastroparesis, and gallbladder complications/events [2,5].

GLP-1RAs have also been shown to increase glomerular filtration rate (GFR), fractional excretion of sodium and potassium, and increase renal blood flow in certain rodent and human studies [6,7]. Hypokalemia, however, is not a reported side effect of semaglutide from prior clinical trial data, and it is not mentioned as a reported side effect in the World Health Organization's VigiBase or in the United States Food and Drug Administration Adverse Event Reporting System.

Hypokalemia as a diagnosis may have a multifactorial origin. Common causes include gastrointestinal losses via diarrhea and/or emesis, decreased oral intake, renal losses, and intracellular shifts [8]. Semaglutide is known to cause diarrhea, emesis, and decreased oral intake; however, hypokalemia is not listed as a common side effect [2,5]. Here, we describe two patients who developed clinically significant hypokalemia resulting in hospitalization while taking semaglutide.

## Case presentation

Case one

A 48-year-old female with a past medical history of type 2 diabetes mellitus, stiff-person syndrome, epilepsy, lupus, and hypothyroidism presented to the clinic for diabetes management. Her initial hemoglobin A1c (HbA1c) was 7.2% (normal reference range: 4.0-5.6%), C-peptide was 5.0 ng/mL (reference range: 1.1-4.4 ng/mL), potassium was normal at 3.8 mmol/L (reference range: 3.6-5.2 mmol/L), and BMI was 32.2 kg/m^2^ with a weight of 96.2 kg. She was started on semaglutide injections for glucose control and weight loss at a starting dose of 0.25 mg weekly for four weeks, followed by 0.5 mg weekly for four weeks, and eventually 1 mg weekly thereafter. At her three-month follow-up, she reported 35 lb (15.9 kg) weight loss. HbA1C was 6.3%, C-peptide was 4.6 ng/mL, and potassium was 3.7 mmol/L.

Six months after drug initiation, the patient developed worsening nausea and emesis. She was reluctant to stop the medication due to concerns of weight regain. She presented to an outside emergency department with potassium at 2.7 mmol/L (reference range: 3.6-5.2 mmol/L). Additional laboratory test results included chloride of 90 mmol/L (reference range: 96-106 mmol/L), serum glucose of 149 mg/dL (reference range: 70-140 mg/dL), normal bicarbonate, normal leukocytes, and creatinine of 0.56 mg/dL (reference range: 0.59-1.04 mg/dL), which was her baseline renal function (Table 1). She received potassium replacement at the outside hospital.

**Table 1:** Laboratory Results From Initial Hospital Admission

Parameter	Results	Normal Reference Range
Potassium	2.7 mmol/L	3.6–5.2 mmol/L
Chloride	90 mmol/L	96–106 mmol/L
Glucose	149 mg/dL	70–140 mg/dL
Bicarbonate	25 mmol/L	20–29 mmol/L
Leukocytes	8.0x10^9^/L	3.4–9.6x10^9^/L
Creatinine	0.56 mg/dL	0.59–1.04 mg/dL

Eleven months after drug initiation, she again reported continued nausea, emesis, minimal food intake, and further weight loss that had been present consistently for the last several months. The patient estimated that, in total, she had lost approximately 100 lb since starting the medication. HbA1C was 5.1%, and potassium was 3.0 mmol/L. Semaglutide therapy was stopped.

Two weeks later, she was readmitted for persistent emesis, decreased oral intake, and hypokalemia with potassium as low as 2.6 mmol/L. Due to several months of continued nausea, emesis, and decreased oral intake, she required peripheral parenteral nutrition and potassium replacement and was transitioned to a feeding tube as she could not support herself nutritionally with oral intake. Her C-peptide was 1.0 ng/mL with a chloride level of 95 mmol/L, glucose of 161 mg/dL, normal bicarbonate, normal venous pH, normal white blood cell count, baseline renal indices, and beta-hydroxybutyrate less than 3.0 mmol/L (reference range: <0.4 mmol/L) (Table 2). Computed tomography scan of the abdomen and pelvis was negative for any acute processes. She was started on low-dose basal-bolus insulin in the hospital, and her feeding tube was removed after one month.

**Table 2:** Laboratory Values From Second Hospital Admission

Parameter	Results	Normal Reference Range
Potassium	2.6 mmol/L	3.6-5.2 mmol/L
Chloride	95 mmol/L	96-106 mmol/L
C-peptide	1.0 ng/mL	1.1-4.4 ng/mL
Glucose	161 mg/dL	70-140 mg/dL
Bicarbonate	24 mmol/L	20-29 mmol/L
Venous pH	7.404	7.320-7.430
Leukocytes	8.5x10^9^/L	3.4-9.6x10^9^/L
Creatinine	0.49 mg/dL	0.59-1.04 mg/dL

**Figure 1:**
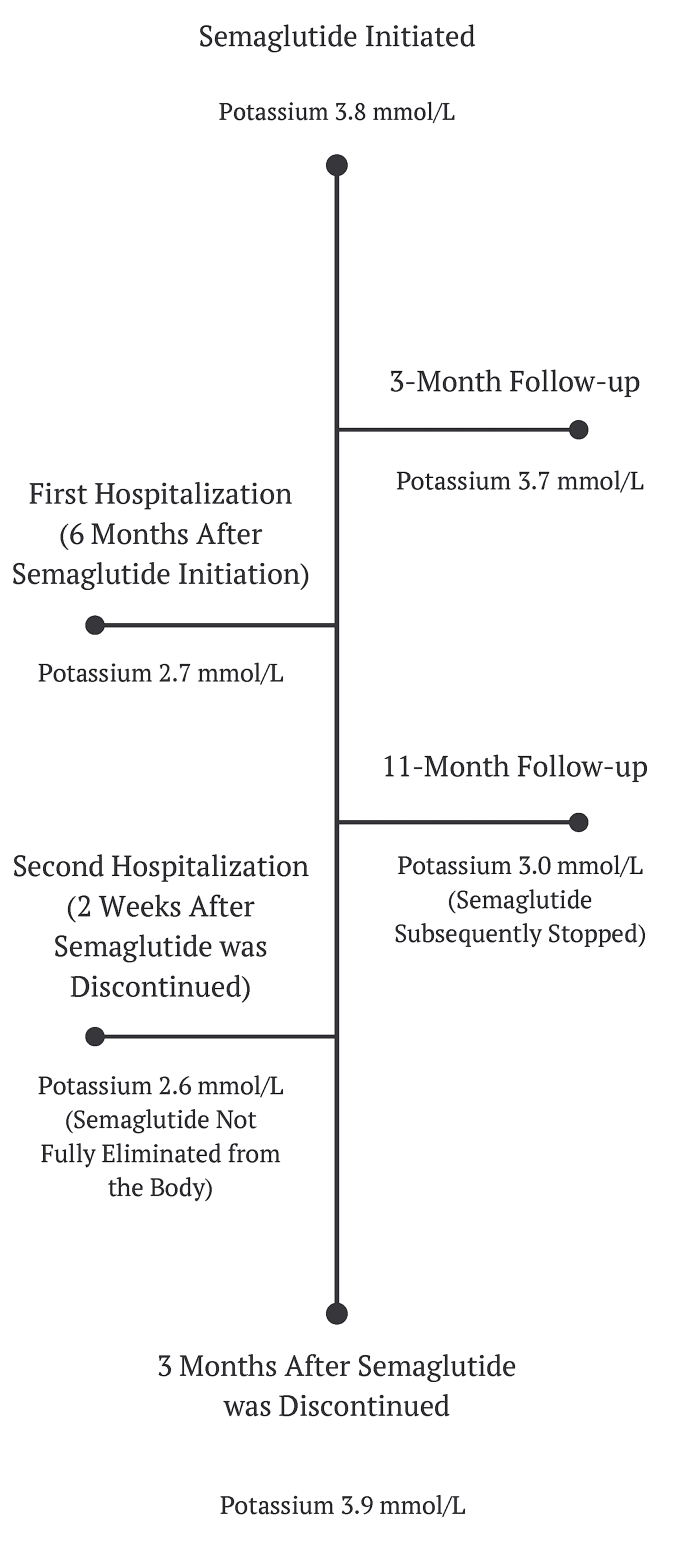
Timeline of Events for Case One Image created by the authors.

The patient has gained 15 lb (6.8 kg) since this hospitalization at her most recent follow-up, and her potassium normalized after discontinuation of semaglutide (Table 3) (Figure 1).

**Table 3:** Potassium and Hemoglobin A1c Trends

Parameter	Initial Visit	At the Three-Month Follow-Up	First Hospital Admission (Six Months After Semaglutide Initiation)	At the 11-Month Follow-Up	Second Hospital Admission (Two Weeks After Semaglutide Was Discontinued but Not Fully Eliminated From the Body)	Three Months After Semaglutide Was Discontinued	Normal Reference Range
Potassium	3.8 mmol/L	3.7 mmol/L	2.7 mmol/L	3.0 mmol/L	2.6 mmol/L	3.9 mmol/L	3.6–5.2 mmol/L
Hemoglobin A1c	7.2%	6.3%	-	5.1%	-	-	4.0–5.6%

Case two

A 45-year-old female with a past medical history of class III obesity, prediabetes, and hypertension presented to the clinic for treatment of obesity. She was started on semaglutide 0.25 mg subcutaneously weekly for four weeks, followed by 0.5 mg weekly for four weeks, with plans to continue up-titration of the dose in four-week intervals as tolerated. The patient reported a weight loss of 36 lb (16.4 kg) in addition to decreased appetite after 1.5 months on semaglutide. Laboratory studies conducted one month prior to medication initiation revealed a potassium level of 3.4 mmol/L (reference range: 3.2-5.1 mmol/L). She was requiring 20 mEq of potassium daily to maintain her potassium on a stable dose of chlorthalidone 50 mg daily for hypertension prior to semaglutide therapy. Hyperaldosteronism was ruled out with outpatient testing.

After 1.5 months of semaglutide 0.5 mg weekly, the patient's serum potassium decreased to 3.1 mmol/L (reference range: 3.6-5.2 mmol/L). This was in the absence of any diarrhea or emesis. Additionally, no other medications were added or adjusted during this period. Repeat potassium five days later was 2.5 mmol/L, which prompted admission to the hospital under observation status. Her creatinine on admission was 0.90 mg/dL, which was her baseline renal status. The patient was asymptomatic. She was treated with a combination of oral and intravenous potassium. A total of 80 mEq of potassium was administered intravenously and 120 mEq orally over the course of her less than 24-hour admission.

**Figure 2:**
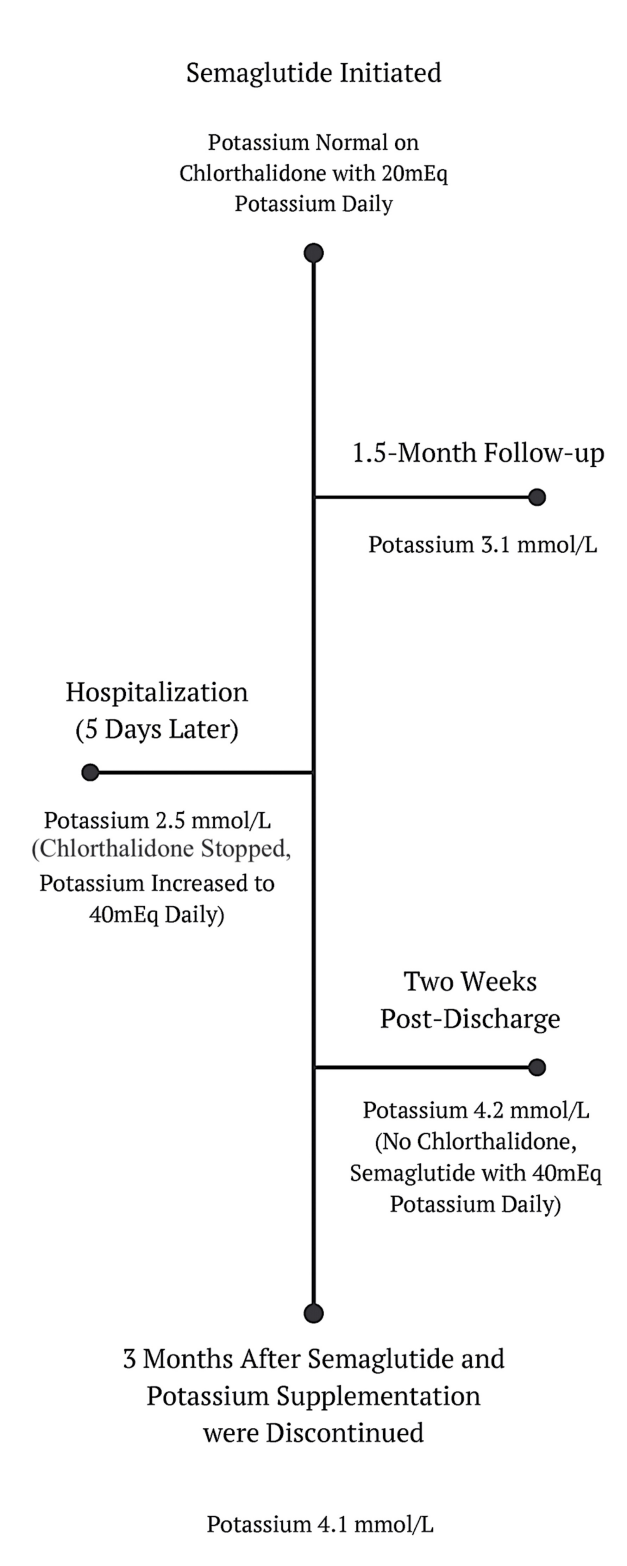
Timeline of Events for Case Two Image created by the authors.

On discharge, the patient was also instructed to discontinue her chlorthalidone that she had been on for several years prior to her episode of hypokalemia. Her home oral potassium was increased to 40 mEq daily. These interventions improved her potassium to 4.2 mmol/L two weeks after hospital admission (Table 4). She required double the amount of oral potassium to maintain her potassium within the normal range while on semaglutide than she did while on chlorthalidone alone. The semaglutide was eventually titrated up to 1.7 mg weekly while still maintaining a potassium level of 40 mEq daily to keep her potassium levels within the normal range. However, shortly after her final dose increase, semaglutide had to be discontinued due to insurance issues. Subsequently, potassium supplementation was also discontinued. While off both potassium supplementation and semaglutide, her serum potassium remained normal at or above 3.8 mmol/L (Table 4) (Figure 2).

**Table 4:** Potassium Level and Potassium Supplementation Requirement Trends

Parameter	Prior to Semaglutide but on Chlorthalidone	On Semaglutide Without Chlorthalidone	Without Semaglutide or Chlorthalidone
Potassium	3.4 mmol/L (normal reference range: 3.2–5.1 mmol/L)	4.2 mmol/L (3.6–5.2 mmol/L)	3.8 mmol/L (3.6-5.2 mmol/L)
Potassium supplementation	20 mEq daily	40 mEq daily	None

## Discussion

These are two separate cases of hypokalemia requiring hospitalization in patients simultaneously taking semaglutide. The first case highlights a patient with months of persistent nausea, emesis, and decreased oral intake that started after initiating semaglutide. She then went on to develop clinically significant hypokalemia requiring hospitalization twice. The patient did not have a leukocytosis during either admission and had persistent symptoms for months, which is inconsistent with a gastrointestinal illness.

The second case highlights a patient on a stable dose of potassium replacement and chlorthalidone who required additional potassium supplementation once initiated on semaglutide. She required continued potassium replacement after stopping chlorthalidone while on semaglutide alone, but no longer required any potassium supplementation after semaglutide was discontinued. She had no other medication adjustments during this time.

Clinically significant hypokalemia as an adverse effect of semaglutide has not been described in large clinical trials as significantly higher than placebo, nor is it listed as an adverse effect in the drug label [9,10]. The mechanisms by which semaglutide may contribute to lower potassium may be multifactorial. One aspect may be related to gastrointestinal losses from nausea, emesis, diarrhea, and dehydration. This, in conjunction with decreased oral potassium intake from daily nutrition, may cause/exacerbate hypokalemia [8].

A secondary cause of hypokalemia associated with GLP-1RAs may be due to their effects on the kidneys. Various studies have characterized the presence of GLP-1 receptors in the renal vasculature and the proximal convoluted tubules [6,11]. GLP-1RAs are also known to increase diuresis, natriuresis, renal blood flow, and GFR in rats [6]. They have also been shown to increase the fractional excretion of potassium in rats and humans [6,7]. This can potentially contribute to hypokalemia observed in patients using semaglutide.

In summary, hypokalemia during semaglutide therapy has not been previously reported in the literature. These two cases do not imply a direct causal link between semaglutide and hypokalemia; however, it is important to note this clinical observation as the use of GLP-1RAs increases. The underlying pathophysiological mechanism for this remains unknown, but it may be multifactorial due to a combination of gastrointestinal losses, decreased oral intake, and/or increased renal excretion of potassium. Further studies are required to investigate the effect of semaglutide on potassium levels and to further characterize the rate of severe hypokalemia in patients taking GLP-1RAs. Physicians should be aware of the possibility of hypokalemia and consider monitoring patients who are at high risk.

## Conclusions

Hypokalemia is not a documented side effect of semaglutide injections. In this report, we describe two patients from our clinical practice who experienced hypokalemia that required hospitalization while taking semaglutide. Possible mechanisms include renal excretion and/or decreased oral intake due to nausea or emesis. Patients and physicians need to be aware of this possibility and monitor for it in patients with additional risk factors for hypokalemia or who are on medications that can cause hypokalemia.
